# Research progress in agitation early warning monitoring and new technologies for critically ill patients: a narrative review

**DOI:** 10.3389/fmed.2026.1915175

**Published:** 2026-08-18

**Authors:** Jintao Wei, Shouyin Jiang, Mengting Yan, Qiang Li, Pengyuan Chen, Zhiying Kang, Shanxiang Xu, Mao Zhang

**Affiliations:** 1 Department of Emergency Medicine, Second Affiliated Hospital, Zhejiang University School of Medicine, Hangzhou, China; 2 Key Laboratory of the Diagnosis and Treatment of Severe Trauma and Burn of Zhejiang, Hangzhou, China; 3 Zhejiang Province Clinical Research Center for Emergency and Critical Care Medicine, Hangzhou, China; 4 National Emergency Medical Rescue Base, Hangzhou, China; 5 Department of Nursing, The Second Affiliated Hospital of Zhejiang University School of Medicine, Hangzhou, China

**Keywords:** agitation, artificial intelligence, computer vision, critical care, deep learning, early warning monitoring, multimodal

## Abstract

Agitation is a common and clinically significant problem in critically ill patients, closely associated with unplanned extubation, device removal, prolonged hospital stay, and increased mortality. Traditional assessment tools such as the richmond agitation-sedation scale (RASS) and sedation-agitation scale (SAS), despite their widespread use, are limited by intermittent assessment, subjectivity, and inability to provide continuous monitoring. In recent years, the rapid development of artificial intelligence and sensing technologies has opened new avenues for objective, continuous, and real-time agitation monitoring through vital sign-based parameters, video surveillance with computer vision, wearable devices, and multimodal fusion approaches. This narrative review systematically examines the epidemiological characteristics and clinical consequences of agitation in critically ill patients, analyzes the limitations of traditional assessment tools, and highlights emerging technologies based on physiological signals, computer vision, deep learning, and multimodal integration. The current status and challenges of agitation monitoring in various clinical settings—including intensive care units, emergency departments, prehospital environments, and inter-hospital transport-are discussed. Finally, future directions for multimodal early warning systems in complex environments are proposed, providing a theoretical foundation for constructing a comprehensive agitation monitoring system spanning prehospital, in-hospital, and transport settings based on vital signs and video-based behavioral analysis.

## 1 Introduction

Critically ill patients are prone to agitation due to factors such as disease stress, sedative medications, the monitoring environment, and sleep deprivation. This manifests as physical activity, anxiety, and aggressive behavior, which interferes with treatment and threatens safety. Studies indicate that patients’ fear of illness, pain, and fluctuations in consciousness can trigger feelings of vulnerability, while a lack of effective communication and increased dependence can exacerbate agitation (1). In the intensive care unit (ICU), agitation is a manifestation of suffering and a clinical alarm; its pathophysiological mechanisms involve neurotransmitter imbalance, brain dysfunction, metabolic abnormalities, and pain stimulation. Pain activates the sympathetic nervous system, inducing or worsening agitation. Delirium is closely related to agitation, particularly hyperactive delirium, which is a life-threatening emergency.

In scenarios such as the ICU, emergency departments, and prehospital emergency care, agitation identification faces even more severe challenges. Traditional assessment tools like the the richmond agitation-sedation scale (RASS), although widely used clinically, suffer from issues such as high subjectivity, poor timeliness, discontinuity, and low accuracy. A survey showed that the proportion of standardized agitation assessments within 24 h of ICU admission was only 29.3% (2). This implementation gap is not an isolated challenge in critical care; across the broader acute care spectrum, Jiang et al. (3) observed that substantial practice variability persists in prehospital emergency settings, and the persistent discrepancy between evidence-based protocols and real-world on-scene care delivery underscores the critical need for standardized, objective assessment strategies adapted to resource-constrained environments. In prehospital emergency settings, limited resources and noisy environments make managing agitation in elderly patients particularly difficult, often leading to ethical dilemmas. These differences highlight the inadequacies of traditional methods and underscore the urgent need for more objective and continuous technologies.

In recent years, new technologies such as wearable devices, artificial intelligence, and computer vision have made real-time, objective monitoring of agitation possible. High-frequency wrist accelerometers can continuously reflect sedation depth and predict increases in RASS scores (4). Quantitative consciousness index (qCON) based on electroencephalography (EEG) can be used to assess sedation levels (5). The bispectral index (BIS), as an adjunct to RASS, can identify deep sedation and reduce medication usage (6). Multimodal deep learning frameworks that integrate EEG with signals such as heart rate and blood pressure can significantly improve assessment accuracy (7). However, these technologies still require solutions regarding algorithm generalization, device portability, data privacy, and clinical integration.

This review covers five core sections: epidemiology of agitation, limitations of traditional assessment tools, advances in emerging early warning technologies, clinical application scenarios, and future research directions. On this basis, we highlight that future research should focus on constructing multimodal early warning models integrating physiological, behavioral and environmental data, carry out prospective validation, and promote the transition from passive response to active early warning.

## 2 Materials and methods

This study is framed as a narrative review. Systematic retrieval was adopted to comprehensively cover research advances in this field, yet full systematic review methodologies [such as quantitative meta-analysis, preferred reporting items for systematic reviews and meta-analyses (PRISMA)-compliant reporting, and research protocol registration] were not implemented.

Relevant literature published from January 2016 to June 2026 was retrieved from the PubMed, Embase, and Web of Science databases, with the retrieval scope limited to the title and abstract fields. The complete search strategy is presented as follows: (“agitation”[Title/Abstract] OR “agitated”[Title/Abstract] OR “psychomotor agitation:[Title/ Abstract] OR “restlessness”[Title/Abstract]) AND (“critical illness”[Title/Abstract] OR “critical care”[Title/Abstract] OR “intensive care”[Title/Abstract] OR “ICU”[Title/Abstract] OR “prehospital”[Title/Abstract] OR “ambulance”[Title/Abstract] OR “interfacility transport”[Title/Abstract]).

The inclusion criteria for analysis were as follows: human studies focusing on early warning, continuous monitoring, automatic recognition technologies for agitation, or applications of related emerging technologies (including artificial intelligence, video-based monitoring, wearable sensors, and multimodal fusion). The exclusion criteria were: non-clinical studies, non-English publications, case reports, editorials, letters, comments, and studies published only as abstracts.

Data extraction and classification were performed on the 36 finally included articles, and a comprehensive analysis was conducted based on their core statistical results (Supplementary Table 1).

## 3 Epidemiological characteristics and clinical hazards of agitation in critically ill patients

### 3.1 Epidemiological characteristics of agitation

Agitation in critically ill patients is a common clinical problem in the ICU, with incidence rates varying depending on the study population, assessment tools, and diagnostic criteria. A prospective cohort study showed that among patients expected to stay in the ICU for more than 48 h, the incidence of agitation was as high as 31.8% (8). Another systematic review and meta-analysis including 10 studies revealed that the pooled prevalence of agitation in adult ICU patients was as high as 55.65% (95% confidence interval [CI]: 40.07%–71.24%), suggesting that more than half of critically ill patients experience agitation during their ICU stay (9). The occurrence of agitation is not random; it exhibits a certain circadian rhythm and is closely related to fluctuations in sedation states. In mechanically ventilated patients, the incidence of agitation is higher and often coexists with delirium. One study pointed out that delirium is an independent risk factor for agitation, with an odds ratio (OR) of 24.14 (CI: 95% 5.15–113.14; *P* < 0.001) (8).

Risk factors for agitation can be categorized into three main types: individual baseline factors, primary disease-related factors, and iatrogenic intervention factors (10, 11). Regarding demographic characteristics, while gender differences are not obvious, the age distribution presents a bimodal pattern. Young and middle-aged patients are often induced to agitation by high-intensity stressors such as trauma and surgery, whereas elderly patients are more associated with underlying conditions like delirium and cognitive decline. A history of underlying diseases, alcohol, or drug dependence is an independent risk factor for agitation, closely related to withdrawal symptoms and stress susceptibility (12). Central nervous system diseases (such as traumatic brain injury and stroke) directly lead to consciousness disorders and abnormal behavioral regulation, with agitation incidence rates exceeding 50%.

Metabolic disorders, infections, and systemic inflammatory response syndrome indirectly induce agitation by interfering with neurotransmitter balance or disrupting the blood-brain barrier. Elderly patients are more prone to agitation due to neurodegenerative changes and cognitive impairment in the central nervous system, but young patients also show a high incidence trend due to vigorous metabolic activity and strong stress responses (13).

**FIGURE 1:**
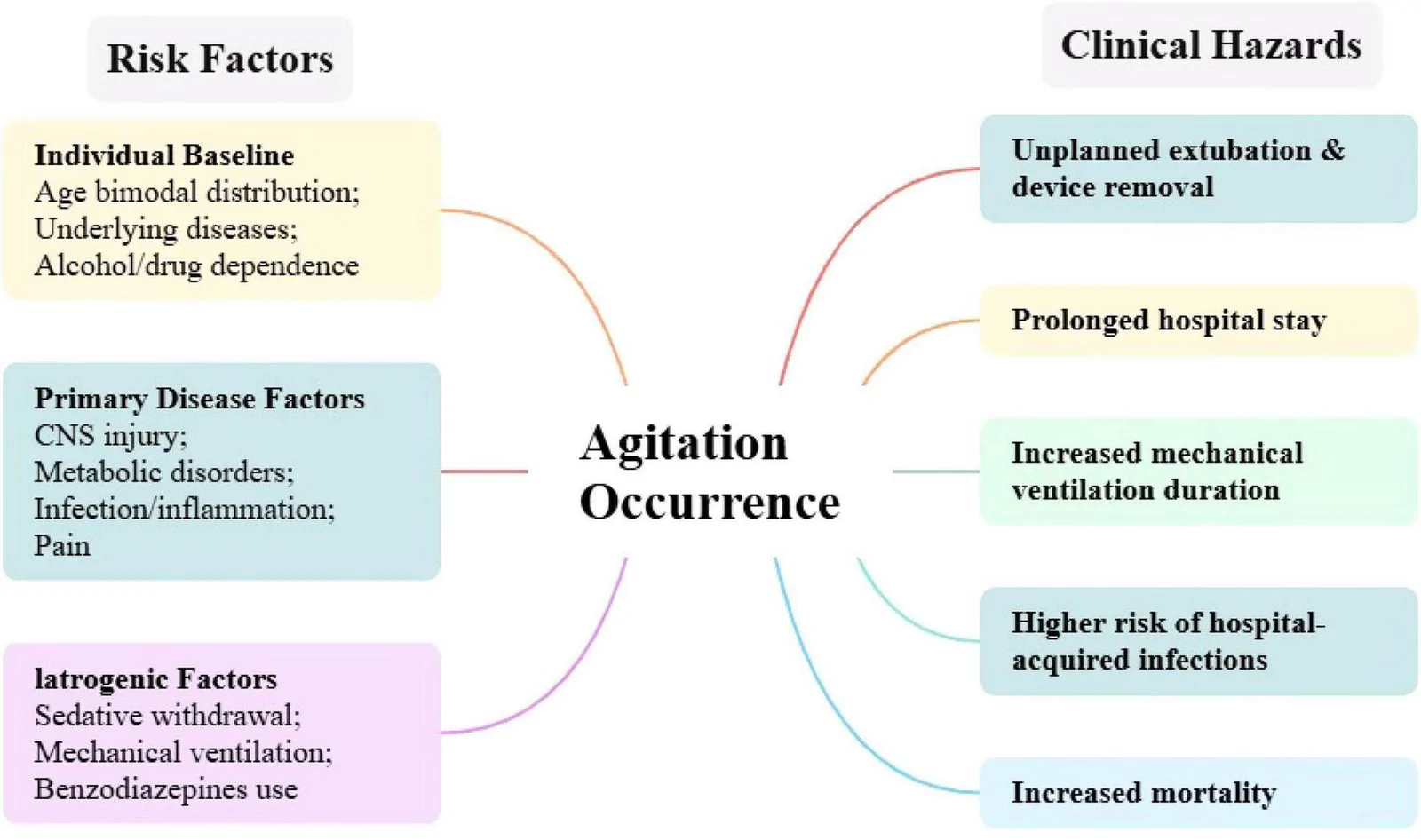
Mechanism diagram of risk factors and clinical hazards of agitation in critically ill patients.

Regarding iatrogenic factors, the duration, dosage, and sudden reduction or discontinuation of sedative and analgesic drugs are key factors inducing agitation, especially drug dependence or withdrawal reactions produced after long-term use. Mechanically ventilated patients are prone to ventilation-related agitation due to airway discomfort, positive end-expiratory pressure stimulation, and mismatched ventilation modes (14). It is believed that these factors may induce agitation by affecting the neurotransmitter balance of the central nervous system or triggering abnormal stress responses. Therefore, identifying these high-risk populations and triggers is crucial for early prevention and intervention (Figure 1).

### 3.2 Adverse events and hazards related to agitation

The harm caused by agitation to critically ill patients is multifaceted and has serious consequences. The most direct hazard is the causation of a series of safety incidents. Among these, accidental extubation is one of the most common adverse events in the ICU, which can affect the airway and even endanger life. Patients in an agitated state cannot cooperate with treatment and often forcibly remove endotracheal tubes, central venous catheters, or arterial catheters due to violent struggling. This not only increases the difficulty and risk of medical procedures but may also trigger severe complications such as massive hemorrhage and pneumothorax. Secondly, persistent agitation seriously interferes with the achievement of analgesia and sedation treatment goals. Agitated patients have fewer days free from mechanical ventilation by the 7th day of ICU admission (8). Prolonged mechanical ventilation itself increases the risk of secondary infections such as ventilator-associated pneumonia and barotrauma. Therefore, precise and effective management of agitation is not only necessary to ensure patient safety but also a key link in improving prognosis.

## 4 Traditional agitation assessment tools and their limitations

### 4.1 Common assessment scales and their characteristics

In the ICU, accurate assessment of patient agitation and sedation levels is a prerequisite for implementing effective management. Currently, the most commonly used clinical assessment tools include the RASS, the SAS and ramsay sedation scale (RSS). All these scales rely on nurses performing subjective scoring through direct observation and interaction with patients. Among them, the RASS scale can be used as an evidence-based tool in conjunction with the awakening and breathing coordination, choice of analgesia/sedation, delirium management, early mobility bundle (ABCDE) bundle and the Critical-Care Pain Observation Tool to reduce ventilation time (15). Clinically, the RASS and the confusion assessment method for the ICU (CAM-ICU) can be combined to assess postoperative delirium (16). The combined use of RASS and CAM-ICU enables twice-daily standardized delirium screening in mechanically ventilated patients, with quantitative EEG studies demonstrating that this dual-assessment approach achieves excellent discrimination between delirious and non-delirious states (area under the curve [AUC] = 0.94, 95% CI 0.83–0.99) (17). Furthermore, the integration of RASS-guided sedation monitoring with CAM-ICU-based delirium detection has been shown to improve pain management and reduce the incidence of oversedation, thereby contributing to shorter mechanical ventilation duration (15). The implementation process and effects of bundled strategies for analgesia, sedation, and delirium management (such as the ABCDE or ABCDEF bundles) have received attention in relevant studies (18) (Table 1).

**TABLE 1:** Comparison of traditional agitation-sedation assessment tools.

Assessment tool	Score range	Assessment method	Key advantages	Key limitations
RASS	−5 to +4	Graded via observation and stimulation	Fine grading, robust evidence, compatible with bundle protocols	Intermittent assessment, subjective, no continuous monitoring
SAS	1 to 7	Graded by response to stimulation	Widely adopted, aligned with clinical management needs	Intermittent subjective assessment, poor sedation depth resolution
RSS	1 to 6	Sedation-focused grading	Simple to operate, historically widespread	Single agitation dimension, insufficient precision

RSS, ramsay sedation scale.

### 4.2 Limitations of traditional assessment tools

Although scales like RASS and SAS are core tools in ICU clinical practice, their inherent limitations cannot be ignored. First, current guidelines related to sedation have limitations regarding the frequency of sedation assessment. Most Polish ICUs do not fully utilize sedation and delirium monitoring tools and fail to follow relevant guidelines (19). Secondly, for patients unable to communicate effectively verbally, such as those under sedation, suffering from dementia, or having consciousness disorders, nurses must rely on a comprehensive interpretation of limb movements, facial expressions, and changes in vital signs when judging agitation. ICU nurses had the lowest correct answer rate on questions related to the interpretation of delirium assessments, suggesting a need to strengthen training on the use and interpretation of assessment tools (20) (Table 1).

## 5 Research progress in new agitation early warning technologies

### 5.1 Early warning technologies based on vital sign parameters

Clinically, agitation episodes are often preceded by significant changes in autonomic nervous system function, providing a physiological basis for early warning technologies based on vital sign parameters. Specifically, the prodromal phase of agitation is often characterized by reduced heart rate variability (HRV), reflecting relatively enhanced sympathetic activity and weakened parasympathetic activity, accompanied by signs such as intensified blood pressure fluctuations and increased respiratory rate. Wearable wireless devices can be used to monitor parameters such as vital signs in ICU patients. For example, High-frequency wrist accelerometers can continuously reflect sedation depth and predict increases in RASS scores. a study targeting ICU patients utilized support vector machine algorithms to extract HRV indicators from electrocardiogram (ECG) signals, successfully distinguishing different sedation levels from “coma” to “agitation,” with personalized model accuracy reaching 67% (21). Additionally, the analgesia nociception index (ANI) based on HRV spectral analysis has been used to assess pain; studies found that when the ANI threshold was set at 42.5, its negative predictive value was as high as 90.4%, indicating high value in excluding significant pain (22).

Multiple studies indicate that HRV frequency domain indicators extracted from ECG signals are significantly correlated with the timing of agitation onset. For instance, the high-frequency power/low-frequency power ratio, a classic indicator reflecting sympathetic-vagal balance, can predict the occurrence of agitation events through its dynamic changes. HRV can be used to distinguish sedation levels in ICU patients (21). A study on patients with dementia also found a characteristic autonomic response pattern before the outbreak of aggressive behavior: initial parasympathetic inhibition (increased heart rate), followed approximately 30 s later by sympathetic activation (increased skin conductance) (23). This further confirms the potential of autonomic nervous system changes as agitation early warning signals. Furthermore, measuring HRV through non-contact photoplethysmography imaging (PPGI) technology also shows application prospects; one study confirmed that PPGI could detect HRV change trends in frail elderly patients during rehabilitation, providing new ideas for non-invasive, continuous monitoring (24).

“Physiological Early Warning Indices” integrating multi-dimensional parameters such as heart rate, blood pressure, respiratory rate, and oxygen saturation have been piloted in some ICUs. This multi-parameter fusion strategy aims to improve the prediction accuracy of agitation events through comprehensive analysis. However, machine learning applications in clinical physiological monitoring still face challenges regarding regulation, law, and data quality. For example, a study on hospitalized COVID-19 patients constructed a delirium prediction model using electronic health record data, achieving a c-index of 0.75 in external validation, indicating that model performance still has room for improvement in complex clinical backgrounds (25). Therefore, future research needs to develop more robust algorithms to effectively distinguish physiological parameter changes caused by the disease itself from specific changes in the agitation prodromal phase, thereby improving the specificity and positive predictive value of early warnings.

In summary, agitation early warning technologies based on vital signs have initially demonstrated value in early identification. However, they suffer from insufficient specificity and limited generalization ability due to interference from confounding factors such as underlying diseases. Moreover, existing studies are mostly single-center retrospective explorations with low evidence strength. Future efforts need to focus on developing highly robust algorithms, deepening multi-parameter fusion, conducting multicenter prospective validations, constructing personalized early warning systems, and promoting clinical translation of these technologies.

### 5.2 Technologies based on video monitoring and computer vision

As a contact wearable solution for behavioral monitoring, high-frequency wrist accelerometers can continuously reflect sedation depth and predict increases in RASS scores (4). Building on the idea of motion-based agitation recognition, non-contact video monitoring empowered by computer vision has emerged as another key technical direction. Using ceiling-mounted cameras or infrared cameras in wards, computer vision algorithms (such as pose estimation, optical flow analysis, and behavior recognition) can extract patient limb movement trajectories and body movement frequencies from video streams. This enables passive, non-contact, continuous monitoring of patient body movements. This technology avoids the discomfort or constraints that physical sensors might cause, making it particularly suitable for ICU patients who require minimal disturbance. One study collecting data via wearable sensors and cameras found significant differences between delirious and non-delirious patients in facial expressions, limb movements, posture, and environmental factors (such as visiting frequency, nighttime lighting, and sound levels), proving the feasibility of autonomous monitoring using non-invasive systems (26).

Deep convolutional neural networks (CNNs) are trained to recognize agitation-related behavioral patterns, such as frequent hand grasping, increased trunk twisting amplitude, and repeated attempts to sit up. For example, a study utilizing deep learning methods classified motion behaviors into five categories based on location and motion sensor data from residents in long-term care facilities, successfully predicting acute events such as falls, delirium, and urinary tract infections, with sensitivity ranging from 0.88 to 0.91 and specificity from 0.71 to 0.88 (27). Additionally, by analyzing sensor data in the homes of dementia patients, machine learning algorithms were able to identify agitation and abnormal behavior patterns with an accuracy of up to 80% (28). These studies indicate that video-based behavior analysis is an effective means of identifying agitation risks.

Despite the huge potential shown by video monitoring technologies, several technical bottlenecks remain in their clinical deployment. Complex lighting conditions (such as at night or against the light), occlusions (such as coverage by blankets or obstruction by medical staff), and distinguishing patients between multiple beds are major challenges. For example, a study on home-dwelling dementia patients pointed out that although remote monitoring technologies hold great potential, data sparsity, unpredictable behaviors, and “weak” labeling from real-world participants are major challenges (29). Future research needs to develop more robust visual algorithms to cope with complex ward environments and explore solutions such as edge computing to reduce reliance on centralized computing power, thereby promoting the clinical application of this technology.

### 5.3 Comprehensive early warning systems based on multimodal fusion and deep learning

Single-modal early warning technologies are susceptible to false positives or false negatives, and their predictive performance is often limited in complex clinical environments. Multimodal fusion integrates physiological signals, video behavioral data, environmental sounds (such as groaning or shouting), and electronic medical record information to construct more robust and accurate agitation early warning models. For example, a pilot study conducted in a real ICU environment utilized ubiquitous sensing and deep learning to achieve autonomous monitoring, analyzing differences in facial expressions, limb movements, and environmental factors between delirious and non-delirious patients (26).

A recently proposed deep learning framework based on temporal convolutional networks (TCN) and attention mechanisms aims to effectively process multimodal time-series data. For example, one study utilized a Random Forest algorithm, combining demographics, comorbidities, medications, surgery, and physiological measurement data, to successfully predict the occurrence of delirium in hospitalized patients, achieving a receiver operating characteristic area under the curve AUC of 0.909 (30). Another study used deep learning methods to automatically track the consciousness level and delirium status of ICU patients using frontal EEG signals, achieving an AUC of 0.80 for delirium detection (31). These studies demonstrate that deep learning models fusing multiple data sources can significantly enhance early warning performance, providing strong support for clinical decision-making.

Under current conditions, the application of multimodal fusion early warning systems still faces numerous bottlenecks. The primary challenge is the precision of time synchronization for multi-source data; sampling frequencies and clocks of different devices (such as monitors, cameras, and microphones) may differ, requiring precise alignment. Secondly, missing values often occur during the collection of data from different modalities (such as sensor detachment or video occlusion); handling these missing values without affecting model performance is a key issue. Furthermore, applying machine learning to clinical monitoring requires processing complex physiological data, integrating electronic health record data, and undergoing rigorous evaluation before clinical application; related directions still require future research. For example, a systematic review pointed out that although machine learning techniques can be used to predict delirium, existing studies are mostly single-center retrospective studies with varying quality (32). Overcoming the aforementioned bottlenecks will be a key step in realizing the transition of multimodal fusion early warning systems from the laboratory to clinical practice.

Overall, the three existing emerging agitation early warning technologies have demonstrated promising application potential, yet obvious methodological limitations persist in current relevant studies. The vast majority of researches are single-center, small-sample retrospective explorations with low levels of evidence. There exists substantial heterogeneity across various studies in terms of patient inclusion criteria, definition of the gold standard for agitation, and validation approaches, which renders the performance indicators of different technologies incapable of direct horizontal comparison. Future researches should conduct further multi-center, prospective external validations and unify evaluation standards to enhance the generalizability and clinical reliability of research findings (Table 2).

**TABLE 2:** Performance comparison of three novel agitation early warning technologies.

Dimension	Vital sign-based early warning	Computer vision-based monitoring	Multimodal fusion deep learning
Core principle	Based on autonomic nerve changes in pre-agitation phase	Pose estimation + behavior pattern recognition	Joint modeling with multi-source data
Key metrics	HRV, ANI, qCON/BIS	CNN, LSTM, human pose estimation	TCN, attention mechanism, hierarchical fusion framework
Monitoring mode	Continuous acquisition via wearables/monitors	Non-contact continuous camera capture	End-edge-cloud collaborative computing
Key strengths	Clear physiological mechanism, easy to integrate with existing devices	Non-contact, no interference with clinical treatment	High warning precision, adaptable to complex scenarios
Main limitations	Low specificity, confounded by underlying diseases	Susceptible to light/occlusion, privacy concerns	Difficult data synchronization, weak model interpretability

The performance data in this table are derived from heterogeneous studies with different designs, varying inclusion populations, and differing gold-standard definitions. These data are intended solely for technical reference purposes and should not be used for direct cross-effectiveness comparisons.

## 6 Status of agitation early warning monitoring technologies in different clinical scenarios

### 6.1 Agitation early warning monitoring in the ICU

The ICU is the primary setting for agitation monitoring and the scenario where early warning technology research and application are most concentrated. Existing research mainly revolves around two technical pathways. The first is real-time analysis based on vital sign parameters from monitors, for example, using consciousness indices derived from EEG (such as qCON, Patient state index, Narcotrend index, etc.) to objectively quantify sedation depth, thereby compensating for the deficiencies of traditional subjective scales (5). One study confirmed that the qCON index can be used to assess the sedation level of mechanically ventilated patients; it correlates significantly with RASS scores, and when the qCON value is 80, the sensitivity and specificity for identifying inappropriate sedation reach 72.67% and 67.42%, respectively (5). Another study compared two EEG-based sedation depth monitors, SedLine^®^ and NeuroSENSE^®^, finding no significant difference in their performance for predicting sedation depth in ICU patients receiving propofol and remifentanil sedation (33).

Additionally, in recent years, non-contact monitoring technologies based on deep learning have gradually increased. In comprehensive ICUs, video-based deep learning monitoring systems have preliminarily verified their feasibility. For instance, Dai et al.’s study collected video data in an ICU environment, where an LSTM model achieved an accuracy of 0.92 and an AUC of 0.91, indicating good application prospects for non-contact automatic agitation classification in ICUs (34). In the ICU environment, standardized monitoring processes, stable network and power supply, and the configuration of dedicated nursing staff provide favorable conditions for deploying these multimodal systems. However, promotion still faces challenges, such as high equipment costs, patient privacy concerns (surveys show patient acceptance is about 60%–70%), and alarm fatigue caused by system false alarms, the latter of which has become a significant clinical issue. A systematic review and meta-analysis pointed out that the pooled prevalence of agitation in ICUs is as high as 55.65%, and high fever (≥37.5 °C) is significantly associated with agitation (OR = 3.24), highlighting the importance of temperature monitoring in agitation management (9).

At the clinical implementation level, the integration of early warning systems faces procedural obstacles. A multicenter randomized before-and-after controlled study by Mistraletti et al. (35) showed that systematic training of medical staff through an e-learning platform can significantly increase the usage rate of pain, agitation/sedation, and delirium assessment tools in ICUs, although the sustainability of training effects needs further evaluation. Walsh et al. developed a sedation quality assessment tool based on process control methodology to track the quality and safety of pain, agitation, and sedation management in ICUs, providing a methodological reference for the clinical integration of early warning systems (36). Therefore, although agitation monitoring and assessment within the ICU represent the most mature scenario, they are still in the research stage and have a long way to go before full implementation. Furthermore, how to balance technological benefits with costs, privacy, and clinical workload remains a core issue to be solved in the future.

### 6.2 Status of agitation early warning monitoring technologies in non-ICU scenarios

#### 6.2.1 Emergency resuscitation area

The emergency resuscitation area is another potential scenario for the application of agitation early warning technologies, but its environmental specificity brings huge challenges. Rapid patient turnover, complex conditions, and high environmental noise make it difficult to continuously execute traditional scale assessments (such as RASS), and assessment reliability is affected by inter-rater variability. Action recognition systems based on video also face difficulties in emergency environments; frequent entry and exit of personnel easily obstruct cameras, affecting recognition accuracy. Additionally, emergency nursing units are characterized by heavy workloads and tight schedules, leaving medical staff with limited time to operate early warning systems specifically, resulting in almost no clinical studies targeting emergency populations with existing technologies. A few exploratory studies have attempted to use portable wearable devices, such as wristband accelerometers, to monitor body movements. A study on patients with traumatic brain injury indicated that actigraphy is feasible for monitoring agitation in ICUs, and agitation levels are significantly higher in agitated patients (37). However, these studies have small sample sizes, and interfering factors in the emergency environment (such as patient movement and family visits) make it very difficult to establish efficient early warning thresholds based on body movement counts. Therefore, agitation early warning technology in emergency resuscitation areas is still in a very early exploratory stage and is far from clinical application.

#### 6.2.2 Prehospital emergency environment

The prehospital emergency environment is one of the most challenging scenarios for the application of agitation early warning monitoring technologies. Patients are in moving, bumping ambulances; cameras and sensors are in a state of vibration, and fluctuating light conditions severely interfere with the collection of physiological signals. For example, signals such as ECG and oxygen saturation are prone to significant deviations.

At the same time, the main focus of emergency personnel is on life support (such as airway management and cardio pulmonary resuscitation), making it difficult to spare attention for the operation and interpretation of additional monitoring equipment. According to surveys, there are currently no mature agitation early warning systems specifically designed for prehospital ambulances. A few studies have attempted schemes combining single-lead ECG with accelerometers for monitoring, but progress has been slow due to unstable real-time data transmission and severe data loss caused by devices falling during bumps. George et al.’s study showed that sedation depth in mechanically ventilated patients during air medical transport is associated with prolonged hospital stays, suggesting that sedation management during transport requires more refined monitoring means (38).

This indicates that in prehospital environments, the focus of monitoring may lean more toward avoiding over-sedation rather than precise agitation early warning. A systematic analysis by Lachs et al. of emergency medical services protocols across US states showed that while 97% of state-level protocols include guidance on agitation management, only 33% provide special considerations for elderly patients (39). The most common modification is halving the sedative dose (27%), while no protocol provides age-specific adjustments for criteria on physical restraint use or drug selection. Another study by Olives et al. (40) described the intubation rates and clinical outcomes of prehospital ketamine treatment for severely agitated patients, suggesting that while ketamine has a high usage rate in prehospital severe agitation, airway risks must be guarded against. Therefore, developing portable early warning devices suitable for prehospital emergency environments with strong anti-interference capabilities, simple operation, and reliable data is a bottleneck urgently needing breakthrough in this field.

#### 6.2.3 Inter-hospital transport scenario

Inter-hospital transport is a high-risk link in the management of critically ill patients. Inter-hospital transport covers three types of scenarios: transfers between multiple campuses of the same hospital, referrals from lower-level hospitals to higher-level ones, and cross-institutional transfers between peer specialized hospitals. This scenario is also another weak link in the current clinical application of agitation early warning technologies. Jahromi et al.’s study showed that structured transport protocols can significantly reduce patient agitation levels and shorten transport time (41). Accompanying personnel for inter-hospital transport are mostly non-ICU medical staff, primarily emergency physicians and nurses, with the team composition centered on junior medical staff. Most of these personnel have not received systematic training in agitation assessment and lack sufficient ability for early identification and risk prediction of agitation. At the same time, external factors such as long-distance transport, time zone fluctuations, and ambient temperature changes may induce patient agitation or exacerbate existing symptoms.

Currently, risk assessment for inter-hospital transport mainly relies on a one-time agitation scale score before transport, unable to achieve dynamic risk tracking during the journey. For example, one study showed that transport time for trauma patients transferred from the emergency department to the ICU was related to prognosis, but it did not involve dynamic monitoring of agitation during transport. However, a recent randomized controlled trial provided new evidence for structured transport protocols. This study randomly assigned 112 hemodynamically stable patients transferred from the emergency department to the ICU into two groups. The intervention group adopted a predetermined protocol including pre-transport coordination, equipment checks, allocation of ICU specialist nurses, and continuous monitoring. Results showed that the agitation level (measured by RASS score) of patients in the intervention group was significantly lower than that of the control group (mean RASS −2.01 vs −2.64, *P* = 0.04), and the transport time was shortened by nearly half (9.4 ± 2.0 min vs. 16.0 ± 4.0 min, *P* < 0.01) (41). This indicates that structured transport protocols themselves can effectively reduce patient agitation, while there are almost no mature application cases of portable multimodal early warning devices in this scenario.

The dilemmas faced include: whether the battery life of devices can support long-distance transport; how devices handle and store data in areas with network interruptions or poor signals; and whether alarm sounds might interfere with ambulance communications or cause unnecessary panic among patient families. Furthermore, patient safety during transport is the primary consideration; any early warning system must not add extra operational burdens or delay transport time. Therefore, developing early warning devices suitable for inter-hospital transport with high integration, long battery life, and offline analysis capabilities is an important direction for future research (Table 3).

**TABLE 3:** Comparison of agitation monitoring status in different clinical scenarios.

Clinical setting	Current monitoring foundation	Technology maturity	Key challenges	Development direction
ICU	Routine intermittent scale assessment + complete monitoring equipment	Most mature, in clinical validation phase	High equipment cost, alarm fatigue, low patient acceptance	EEG adjunct + video monitoring, evolving toward multimodal fusion
Emergency resuscitation area	Mainly visual observation, rare standardized assessment	Early exploratory stage	Rapid patient turnover, frequent occlusion, hard to sustain monitoring	Small-scale pilot of wrist-worn wearable devices
Prehospital emergency care	No standardized monitoring, relies on staff experience	Nearly blank	Motion artifacts, unstable transmission, high operational burden	Exploration of single-lead ECG + accelerometer solutions
Inter-hospital transport	Single pre-transport assessment, basic vital sign monitoring en route	Blank stage	High battery demand, offline computing requirement, staff capacity gap	Structured transport protocols + 5G portable multimodal device R&D

**FIGURE 2:**
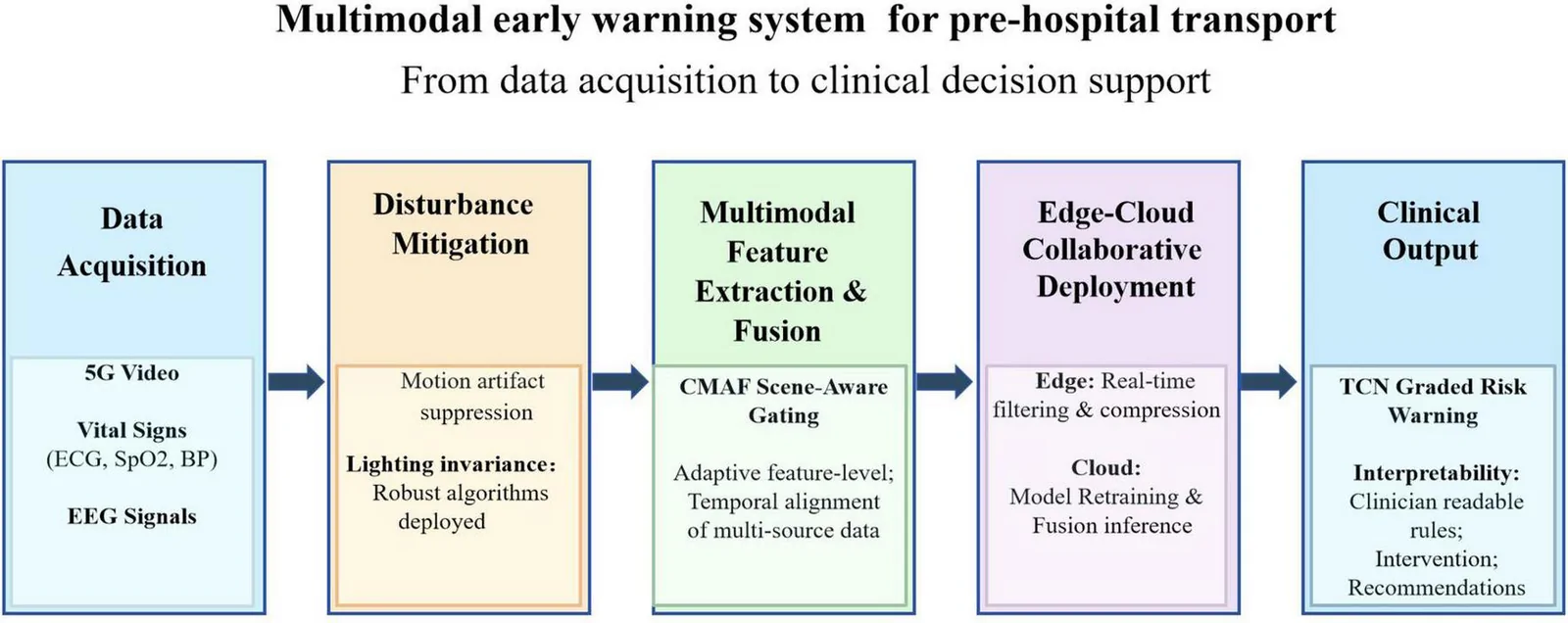
Multimodal early warning system for prehospital transport.

Based on this, our team is currently conducting research on agitation early warning for sedated patient transport in the complex “prehospital to inter-hospital” environment. This study collects video of patients in a sedated state overlaid with vital signs (blood pressure, heart rate, oxygen saturation, etc.) using 5G cameras. Using RASS as the gold standard, this study utilizes the aforementioned data combined with deep learning models for training, aiming to construct a multimodal agitation early warning system capable of adapting to dynamic environmental changes during transport. This system will focus on solving data collection and processing challenges when devices face bumps and signal instability, and explore how to achieve real-time assessment and early warning of agitation risks without increasing the burden on medical staff, thereby providing new solutions for this blank area of technology application (Figure 2).

## 7 Future research directions and development prospects

### 7.1 Construction of multimodal early warning systems in complex environments

Drawing on prior research findings and our team’s practical experience in clinical agitation monitoring, we propose a research scheme “Multimodal Sedation-Agitation Spatiotemporal Evolution Prediction and Interpretable Early Warning System for Complex Prehospital Transport Scenarios.”

### 7.2 Closed-loop management from early warning to intervention

**FIGURE 3:**
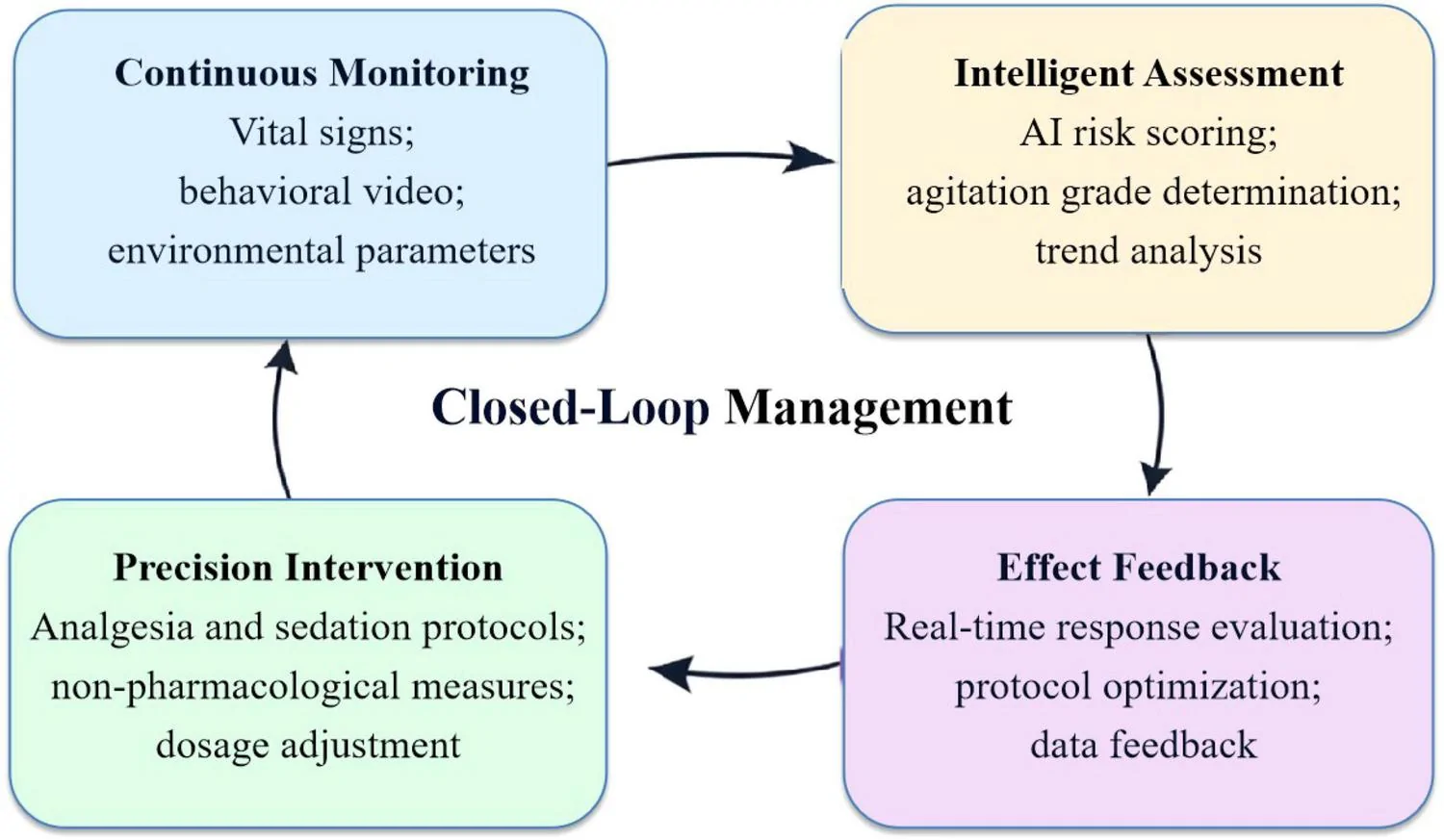
Closed-loop agitation management system.

Constructing a closed-loop management system from early warning to intervention is key to achieving effective agitation management. When a multimodal early warning system identifies that a patient is at risk of agitation or has already become agitated, the system should be able to automatically trigger or assist clinical decisions, forming a “monitoring-early warning-intervention-feedback” closed loop. First, early warning signals should guide clinicians to quickly assess and handle potential triggers of agitation, such as pain, delirium, and mechanical ventilation, among which pain, delirium, and mechanical ventilation have been confirmed as independent risk factors for agitation in ICU patients (8, 42). Existing studies have explored the correlations between inflammatory markers and delirium/coma, providing pathophysiological evidence for stratification of agitation risks (43). For example, for agitation caused by delirium, non-pharmacological intervention strategies should be prioritized, such as early mobilization, reducing sensory deprivation, and improving sleep hygiene, while benzodiazepines should be used cautiously as they may worsen delirium (44, 45). Secondly, closed-loop management should integrate sedation and analgesia protocols. Guideline-based bundled strategies, such as the ABCDEF (A2F) bundle, have been proven to improve patient outcomes by increasing adherence, including reducing the duration of delirium and coma, shortening hospital stays, and improving survival rates (46). Regarding pharmacological intervention, for hyperactive delirium and agitation, valproic acid, as an adjunctive treatment, has been proven to safely reduce the use of combined psychoactive drugs and may lower the risk of polypharmacy (47). Additionally, non-pharmacological interventions such as using valerian and lemon balm extracts to assist quetiapine have also shown potential in reducing agitation and shortening ICU stays (48). Finally, the closed-loop system should continuously monitor intervention effects and dynamically adjust treatment plans based on the patient’s real-time status, for example, through the pairing of daily sedation interruption with spontaneous breathing trials (SBT) to assess the possibility of weaning patients from sedation and ventilators, thereby avoiding over-sedation and prolonged mechanical ventilation (49) (Figure 3).

### 7.3 Interpretability and clinical trust

Although deep learning models show great potential in agitation early warning, their “black box” nature may hinder trust and adoption by clinicians. Therefore, improving model interpretability is an important direction for future research. Interpretability aims to let clinicians understand why a model makes a specific prediction; relevant research includes multimodal framework studies for sedation monitoring in ICU patients, integrating EEG, blood pressure, heart rate, and oxygen saturation signals (7). A feasible method is to adopt attention mechanisms or feature importance analysis to highlight input features that contribute most to the model’s decision. For example, in the hierarchical multimodal fusion with dynamic correction framework, the dynamic correction module fuses neural representations through a confidence-weighting mechanism (7). Furthermore, model interpretability should be combined with clinical knowledge. For instance, models should be able to identify specific physiological patterns related to delirium, such as the elevation of inflammatory markers Interleukin-6 (IL-6), IL-8, tumor necrosis factor-α being associated with the occurrence of delirium the next day (50), or subtle behavioral manifestations related to subclinical seizures, such as ventilator asynchrony and purposeless stereotyped upper limb movements (51).

By associating the model’s prediction results with these known pathophysiological mechanisms, clinicians’ trust in early warning information can be enhanced. Simultaneously, developing visualization tools to present the physiological and behavioral signals focused on by the model to clinicians in an intuitive manner, such as highlighting abnormal waveforms or behavioral segments on bedside patient monitors, will help transform the early warning system from an auxiliary tool into a reliable partner for clinical decision-making.

### 7.4 Full-chain integration of prehospital, in-hospital, and transport

The management of agitation in critically ill patients should not be limited to the ICU ward but should extend to a full-chain integration covering prehospital emergency care, in-hospital transport, and transfers between different departments. This literature is a cohort study on risk factors for agitation in patients admitted to the ICU, mentioning that RASS scores can be used to define agitation within the ICU (8). During in-hospital transport, such as from the emergency department to the ICU or from the ICU to examination departments, patients face environmental changes, handling, and operational stimuli, making it a high-incidence period for agitation and delirium. Studies show that stimulus-induced non-convulsive seizures occur frequently during nursing operations, medical procedures, and family visits, and their clinical manifestations may be merely subtle agitation or ventilator asynchrony (51).

Therefore, during transport, portable multimodal monitoring devices (such as wearable EEG and heart rate variability monitors) should be equipped, and lightweight early warning models should be used for real-time risk assessment. Additionally, full-chain integration also requires establishing unified communication and handover standards. For example, CAM-ICU and intensive care delirium screening checklist are validated ICU delirium assessment tools recommended for routine use (44, 52). For critically ill patients, longer duration of hypoactive delirium is independently associated with long-term cognitive deterioration (53). Ultimately, by integrating data from prehospital, in-hospital, and transport links, an agitation risk prediction model covering the patient’s entire acute disease course can be constructed, achieving a shift from passive response to active prevention, thereby improving overall patient prognosis.

### 7.5 Ethical and privacy legal framework

The continuously exported physiological and video data from monitoring fall under highly sensitive health information and are subject to stringent domestic and international regulations. China’s Personal Information Protection Law mandates that separate consent must be obtained for the processing of sensitive personal information, yet mobile medical privacy policies show a low compliance rate (an average of 67.30%) (54, 55). The EU’s GDPR imposes strict requirements on the secondary use of health data, with inconsistent enforcement standards across member states and insufficient legal certainty (56). Furthermore, ICU privacy covers six dimensions including physical, informational and psychological privacy, and there exists an inherent ethical conflict between continuous video monitoring and the protection of patient privacy (57, 58). While health information sharing facilitates decision-making, it carries risks of sensitive information leakage (59). Meanwhile, the application of new technologies faces a triple dilemma of poor data quality, inadequate privacy safeguards and a lack of certification, accompanied by prominent ethical risks. Most ICU patients suffer from impaired decision-making capacity, and deferred consent serves as a viable alternative for low-risk research with high clinical recognition and overall consent rates (60, 61). Nevertheless, major practical obstacles include the difficulty of legal representatives to arrive on-site and inaccurate prediction of patients’ wishes, which calls for improved supporting mechanisms for legal representation and deferred consent (62, 63).

## 8 Conclusion

As a common dangerous syndrome in critically ill patients, the construction of an agitation early warning monitoring system has shifted from “passive recording” to “active prediction.” The intermittency and subjectivity of traditional assessment tools have exposed obvious limitations in the high-intensity, fast-paced environment of critical care, spawning new early warning technologies represented by vital sign parameters, video monitoring with computer vision, and multimodal fusion with deep learning. Cutting-edge technologies such as AI pose estimation and deep learning multimodal fusion frameworks have shown good application prospects in ICU environments. However, research on agitation early warning in complex environments such as prehospital emergency care and inter-hospital transport is still in its infancy. Future research should focus on constructing multimodal early warning systems adapted to complex environments, achieving closed-loop management from early warning to intervention, and enhancing clinical trust through interpretable AI. A full-chain agitation monitoring system covering prehospital, in-hospital, and transport based on vital signs and video behavior analysis is expected to become an important breakthrough direction for the safety management of critically ill patients.
