# Supplementary material for: Research progress in agitation early warning monitoring and new technologies for critically ill patients: a narrative review
Source: Front Med (Lausanne). 2026 Aug 18;13:1915175. doi: 10.3389/fmed.2026.1915175 (PMC13532560; doi:10.3389/fmed.2026.1915175)
Supplement: Supplementary file 1 [file Table_1.docx]

**Supplementary materials**

**Table 1.Summary table of literatures related to agitation**

|  | **First author** | **Publication year** | **Study design** | **Study population** | **Technology /assessment tool** | **Sample size** | **Key performance indicators** |
| --- | --- | --- | --- | --- | --- | --- | --- |
| 4 | Weeden M | 2020 | Prospective observational pilot study | Adult trauma patients admitted to a tertiary intensive care unit in Melbourne, Australia | High-frequency bilateral wrist accelerometry (100 Hz sampling, Smart Sensor Watch G2 device) | 20 patients (total 160 hours of continuous recording) | 1. The 2-minute moving window of acceleration amplitude variance was proportional to the RASS score; 2. Accelerometry variance spiked immediately before ≥2-point increases in RASS score; 3. Consistent positive correlation between accelerometry variance and RASS across the full score range (-4 to +4) |
| 5 | Harsha MS | 2022 | Prospective observational study | Mechanically ventilated critically ill patients aged 18–60 years receiving standardized propofol-fentanyl sedation in the ICU | Quantium Consciousness Index (qCON, processed EEG monitoring via CONOX monitor) | 87 patients (total 1,218 paired qCON-RASS readings) | 1. Spearman correlation ρ = 0.288 (p < 0.0001) between qCON and RASS; 2. AUC = 0.738 for differentiating minimal sedation (RASS 0 to -1) from moderate sedation (RASS -2 to -3); 3. qCON cutoff of 80 yielded a sensitivity of 72.67% and a specificity of 67.42% |
| 8 | Almeida TM | 2016 | Single-center prospective cohort study | Adult critically ill patients with predicted ICU stay > 48 hours, admitted within 24 hours of ICU admission | Richmond Agitation-Sedation Scale (RASS, score ≥ +2 defined as agitation); CAM-ICU; numerical pain analog scale | 113 | Incidence of agitation: 31.8%; independent risk factors for agitation: delirium (OR=24.14, 95%CI 5.15–113.14, *P*<0.001), moderate/severe pain (OR=5.74, 95%CI 1.73–19.10, *P*=0.004), mechanical ventilation (OR=10.14, 95%CI 2.93–35.10, *P*<0.001), smoking habit (OR=4.49, 95%CI 1.33–15.17, *P*=0.015) |
| 9 | Asmare TB | 2026 | Systematic review and meta-analysis | Adult critically ill patients admitted to intensive care units | Multiple agitation assessment scales (RASS, Riker ASS, SAS, etc.) across included studies | 1306 patients from 10 included studies | Pooled prevalence of agitation: 55.65% (95%CI 40.07–71.24); hyperthermia (>37.5°C) was significantly associated with agitation (pooled adjusted OR=3.24, 95%CI 1.51–4.91, *P*<0.0002) |
| 10 | Leong AY | 2025 | Systematic review and meta-analysis | Adult critically ill patients admitted to ICUs | Delirium screening tools (CAM-ICU, ICDSC); pain assessment tools (CPOT, NRS) | 183,285 patients from 213 original studies | Pooled delirium prevalence: 35.7% (95%CI 32.4–39.0%); hypoactive delirium prevalence: 16.5% (95%CI 12.1–21.4%); pooled delirium incidence: 28.8% (95%CI 23.2–34.8%); pooled pain prevalence: 43.5% (95%CI 28.6–58.9%) |
| 11 | Seeger S | 2026 | Single-center retrospective cohort study | Adult COVID-19 patients requiring ≥72 h invasive mechanical ventilation (IMV) and/or ECMO support in a cardiothoracic ICU | CAM-ICU; Richmond Agitation-Sedation Scale (RASS); structured chart review | 102 (58 in ECMO group, 44 in IMV-only group) | Overall delirium prevalence: 53.9%; delirium rate in IMV-only group: 63.6%; delirium rate in ECMO group: 46.6% (*P*=0.065); independent predictors of delirium: absence of ECMO (OR=3.725, *P*=0.020), male sex (OR=4.740, *P*=0.008), longer IMV duration (OR=1.027, *P*=0.036) |
| 12 | Arroyo-Novoa CM | 2020 | Prospective exploratory observational study | Adult trauma ICU patients receiving opioids and/or benzodiazepines for ≥ 5 days | Customized opioid/benzodiazepine withdrawal syndrome checklist; RASS; CAM-ICU | 50 | Incidence of probable withdrawal syndrome: 44%; agitation prevalence in probable withdrawal group: 41% vs 11% in non-withdrawal group; each 1-point increase in RASS score was associated with higher odds of withdrawal (OR=4.13, 95%CI 2.09–8.16, *P*<0.0005); delirium was associated with withdrawal syndrome (OR=2.69, 95%CI 1.01–7.14, *P*=0.047) |
| 13 | Torres-Contreras CC | 2019 | Prospective cohort study | Adult ICU patients recruited within 24 h of admission, with RASS score ranging from -3 to +4 | RASS; Spanish version of PRE-DELIRIC scale; CAM-ICU | 134 | Cumulative incidence of delirium: 20.2%; proportion of hypoactive delirium: 66.7%; independent risk factors for delirium: infection (RR=3.8, 95%CI 1.6–9.1), age > 60 years (RR=3.2, 95%CI 1.2–8.3); mortality rate in delirium patients: 51.85% |
| 14 | Kerber K | 2020 | Retrospective cohort study | Adult mechanically ventilated patients admitted to the medical intensive care unit (MICU) | Confusion Assessment Method for the Intensive Care Unit (CAM-ICU); Richmond Agitation-Sedation Scale (RASS) | 171 (final analytical sample) | 65% of unplanned extubation events were preceded by delirium; hypoactive delirium accounted for 74% of delirium cases before unplanned extubation; OR for unplanned extubation in COPD patients: 5.2 (*P*<0.05); OR in patients with failed spontaneous breathing trial: 12.6 (*P*<0.01) |
| 15 | Bardwell J | 2020 | Before-after observational study | Adult mechanically ventilated patients in a 34-bed teaching hospital ICU (USA, excluding brain injury, terminal illness and home ventilator-dependent patients) | ABCDE bundle implementation; CPOT for pain assessment; RASS for sedation assessment; RSBI for weaning evaluation | 50 patients total (16 pre-intervention, 34 post-intervention) | Mean ventilation time decreased by 49.5% (from 3.92 to 1.94 days, *P* = .02); mean sedation time decreased by 47.6% (from 3.68 to 1.75 days, *P* = .12); 24-hour reintubation rate 2.9%; nursing staff compliance rate 76.5% |
| 16 | Qiu J | 2025 | Prospective observational study | Postoperative patients admitted to a tertiary hospital SICU in Shanghai, China | CAM-ICU for delirium diagnosis; RASS for sedation/agitation grading; MRC score for ICU-AW assessment; bivariate logistic regression analysis | 402 patients | Incidence of ICU-AW: 30.10%; incidence of POD: 22.89%; hypoactive POD independently associated with ICU-AW (OR = 4.241, 95% CI: 1.490–12.072); POD duration independently associated with ICU-AW (OR = 2.649, 95% CI: 1.422–4.935) |
| 17 | Williams Roberson S | 2023 | Prospective observational cohort study | Mechanically ventilated adult ICU patients with acute respiratory failure due to cardiogenic/septic shock or neurologic failure associated with trauma | 24-hour continuous quantitative electroencephalography (qEEG) with spectral analysis | 25 patients (28 enrolled initially) | AUC of the composite delirium score = 0.94 (95%CI 0.83–0.99); AIC of Clinical + qEEG model = 36 vs 43 for clinical model alone (p=0.006) |
| 18 | Moraes FDS | 2022 | Systematic review and meta-analysis | Adult critically ill patients admitted to ICUs | Systematic literature retrieval; random-effects meta-analysis; qualitative synthesis of implementation barriers and facilitators | 20 studies included, 31,604 total participants | Bundle implementation reduced delirium incidence (OR = 0.15, 95% CI: 0.09–0.26); shortened mechanical ventilation duration and ICU length of stay; reduced ICU and hospital mortality; increased early mobilization rate |
| 19 | Kotfis K | 2017 | Cross-sectional postal survey | Heads/clinical directors of adult ICUs across Poland | Nationwide structured postal questionnaire survey | 165 ICUs responded (37.8% response rate from 436 targeted ICUs) | Sedation monitoring tools used in 46.1% of ICUs; delirium monitored in only 11.9% of ICUs; 19.4% of ICUs had written sedation protocols; 32.1% did not practice daily sedation interruption; haloperidol used for delirium treatment in 77.6% of units |
| 20 | Mu-Hsing Ho | 2022 | Descriptive cross-sectional study | Critical care nurses from ICUs in Taiwan | Web-based 16-item MCQ delirium knowledge quiz; four-domain knowledge assessment | 324 valid responses from critical care nurses | Mean total knowledge score: 10.46/16; correct response rate for assessment & detection domain: 41.5%; CAM-ICU interpretation item correct rate: 30.9%; working experience positively correlated with knowledge score (*r* = 0.178, *p* < .001) |
| 21 | Nagaraj SB | 2017 | Multicenter pilot study; diagnostic algorithm development with leave-one-subject-out cross-validation | Mechanically ventilated adult ICU patients receiving sedative medications | Support vector machine (SVM) classifier using heart rate variability (HRV) features extracted from routine ECG recordings | 70 patients; 21,912 hours of ECG data; 4,258 RASS assessments | Four-class sedation classification: patient-independent accuracy = 59%; patient-specific accuracy = 67%. Binary light vs. deep sedation discrimination: accuracy = 75%, AUC = 0.73, sensitivity = 66%, specificity = 86% |
| 22 | G. Chanques | 2017 | Prospective diagnostic accuracy study | Critically ill, non-comatose, non-communicative adult ICU patients (RASS > −4) undergoing routine care procedures | Analgesia Nociception Index (ANI); both mean ANI (ANIm) and instant ANI (ANIi) derived from HRV spectral analysis | 110 patients; 969 paired pain assessments | ANIi: AUC = 0.73 (95% CI 0.68–0.77). At threshold of 42.5: sensitivity = 61.4%, specificity = 77.4%, PPV = 37.0%, NPV = 90.4%. ANIm: AUC = 0.57 (poor discriminative performance) |
| 23 | Deutsch CK | 2021 | Observational case series / short communication | Dementia inpatients with acute combative behavioral outbursts in a geriatric psychiatry unit | Wearable wristband biosensor monitoring skin conductance, heart rate, and 3-axis accelerometry | 7 participants observed over 200+ hours; 2 captured combative outbursts from 2 patients | Characteristic autonomic pattern identified: immediate parasympathetic withdrawal (heart rate elevation) followed by sympathetic activation (skin conductance increase) with a latency of 27–35 seconds. No advance warning signal detected before outbursts |
| 24 | Yu X | 2022 | Pilot observational cohort study | Frail geriatric inpatients receiving early geriatric rehabilitation, and healthy community-dwelling older adults | Non-contact photoplethysmography imaging (PPGI) for HRV measurement, with reference ECG | 17 analyzable participants (9 community-dwelling, 8 geriatric patients); 3 excluded due to newly detected atrial fibrillation | Group-level HRV tracking: PPGI detected a 24.8% median reduction in LF/HF ratio after rehabilitation, concordant with ECG reference. Individual-level trend tracking: 58.8% of participants achieved Spearman’s ρ = 1.0 between PPGI and ECG |
| 25 | Castro VM | 2021 | Retrospective cohort study; development and external validation of a predictive model | Hospitalized adult patients with laboratory-confirmed COVID-19 across 6 academic and community hospitals | L1-penalized logistic regression (Lasso) machine learning model based on electronic health record (EHR) data | 2907 patients total; 2152 in training cohort, 755 in external validation cohort | External validation: C-index (AUC) = 0.75 (95% CI 0.71–0.79); lift of 2.1 in the top risk quintile. At 80% sensitivity: specificity = 56%, NPV = 92%, PPV = 30%. Optimal cut-point (0.15): sensitivity = 0.73, specificity = 0.69 |
| 26 | Davoudi A | 2019 | Prospective pilot observational study | Critically ill adult patients in surgical ICU, including hyperactive subtype delirious patients and non-delirious patients | Pervasive sensing system (wearable accelerometers, video monitoring, light sensor, sound sensor); deep learning models (MTCNN, FaceNet, OpenFace, multi-person pose estimation) | 22 patients recruited, 17 included in final analysis; 4 persistently delirious patients, 8 persistently non-delirious patients for comparative analysis | Face detection mAP = 0.94; face recognition overall mAP = 0.80; posture recognition F1-score = 0.94; all 15 facial action units showed significant differences between groups (p < 0.01); nighttime sound pressure level and light intensity significantly differed between groups (p < 0.05) |
| 27 | Ramazi R | 2022 | Longitudinal observational study | Older adults with cognitive impairment or dementia in Department of Veterans Affairs long-term care facilities | Real-time locating system (RTLS) with UWB RFID wrist tags; deep temporal clustering; CART decision tree prediction model | 23 residents, 81,323 observational data points, up to 18 months of continuous monitoring | Fall prediction: sensitivity = 0.88, specificity = 0.80; Delirium prediction: sensitivity = 0.90, specificity = 0.84; UTI prediction: sensitivity = 0.91, specificity = 0.71; clustering Silhouette score = 0.67 |
| 28 | Enshaeifar S | 2018 | Longitudinal observational study with machine learning algorithm development | Community-dwelling patients with mild to moderate dementia receiving home care | In-home IoT sensor array (PIR sensors, motion sensors, pressure sensors, door sensor, energy monitor) + physiological monitoring devices; Markov chain model, entropy rate analysis, Hidden Markov Model, hierarchical decision fusion | 12 participants for macro-level pattern analysis; 74 verified agitation/irritability/aggression (AIA) events for micro-level detection | Macro anomaly detection sensitivity = 74%; correlation between detected anomalies and validated notifications = 87%; AIA detection decision fusion AUC = 0.87, weighted precision = 0.81, weighted recall = 0.83, weighted F1-score = 0.80 |
| 29 | Alam R | 2019 | Transdisciplinary prospective observational study with predictive model development | Persons with mild to severe dementia and their family caregivers (dyads) in residential home settings | Wrist-worn Pebble smartwatch with 3-axis accelerometer; Multiple-Instance Learning (MIL) models (axis-parallel rectangle, MI-SVM, MIL-Boost) | 10 dementia-caregiver dyads, 30 days of continuous monitoring per dyad | MIL-Boost: accuracy = 0.87, AUC = 0.92; MI-SVM: accuracy = 0.83, AUC = 0.88; APR: accuracy = 0.76, AUC = 0.71; MIL models outperformed single-instance learning models in F-score and AUC |
| 30 | Corradi JP | 2018 | Retrospective observational cohort study | Adult inpatients with length of stay ≥ 48 h at Hartford Hospital; including both ICU and non-ICU subgroups, with available CAM delirium assessments and longitudinal vital sign data | Distributed Random Forest (DRF) machine learning classifier, with Platt scaling calibration; features cover demographics, comorbidities, medications, procedures and physiological parameters from EHR | Total 64,038 inpatient visits (80% training set: 51,240 visits; 20% validation set: 12,798 visits) | Validation set: • ROC AUC = 0.909 (95% CI: 0.898 to 0.921) • PR AUC = 0.604 • At F2-maximizing threshold: precision = 0.357, sensitivity (recall) = 0.698, specificity = 0.927 |
| 31 | Sun H | 2019 | Single-center prospective observational study | Mechanically ventilated adult ICU patients | Deep learning (CNN + LSTM) based on frontal EEG signals | 174 patients; 3366 RASS assessments, 121 patients with 258 CAM-ICU assessments | RASS tracking: AUC 0.83 (95% CI 0.81–0.85); exact RASS accuracy 24%, 70% accuracy when allowing 1-level difference Delirium detection: AUC 0.80 (95% CI 0.73–0.86); optimal operating point: sensitivity 0.69, specificity 0.83 |
| 32 | Chua SJ | 2021 | Systematic review and meta-analysis | Hospitalized adult inpatients (excluding pure ICU, ED and palliative care settings) | Data mining / machine learning (random forest, support vector machine, artificial neural network, etc.) | 6 unique studies, 178,091 total participants (17,574 delirium cases) | Random forest: AUROC 0.78–0.91; sensitivity 0.59–0.81; specificity 0.73–0.92 |
| 33 | Meerts S | 2026 | Single-center prospective comparative observational study | Critically ill adult ICU patients sedated with propofol (with or without remifentanil / morphine) | Two EEG-based depth-of-sedation monitors: SedLine (PSI index) and NeuroSENSE (WAVCNS index) | 30 patients, 180 data points | Prediction probability (PK): SedLine PSI: 0.826 ± 0.034 NeuroSENSE right hemisphere: 0.809 ± 0.032 NeuroSENSE left hemisphere: 0.797 ± 0.033 No statistically significant difference between the two devices |
| 34 | Dai PY | 2024 | Retrospective model development study | Adult ICU patients with RASS score ≥ -2 | Video streaming motion detection + deep learning (LSTM, random forest, threshold method; MediaPipe pose estimation) | 61 patients; 427 30-second segments, 6405 2-second segments | LSTM model: accuracy = 0.92, AUC = 0.91 |
| 35 | Mistraletti G | 2017 | Multicenter, randomized, before-and-after pragmatic study | Physicians and nurses in general ICUs | E-learning continuous medical education platform for standardized assessment tools (RASS, CAM-ICU, BPS) | 8 ICUs, 374 medical and nursing staff | After training: Validated pain assessment rate increased from 38.1% to 92.9% (P<0.01) Validated delirium assessment rate increased from 0% to 78.6% (P<0.01) |
| 36 | Walsh TS | 2016 | Multicenter observational study | Mechanically ventilated adult ICU patients | Process control methodology; Sedation Quality Assessment Tool (SQAT) | 8 ICUs, 825 patients, 12,385 12-hour care periods | Agitation-sedation domain: Spearman ρ = 0.75 (vs. RASS); inter-rater reliability κ = 0.66 (clinician-clinician) Optimum sedation rate: 45%–70% across ICUs Excessive sedation rate: 12%–38% across ICUs |
| 37 | Saavedra-Mitjans M | 2024 | Prospective single-center observational cohort study | Adult patients with acute traumatic brain injury (TBI) admitted to ICU | Wrist actigraphy (Actiwatch-Spectrum) for rest-activity cycle monitoring | 25 patients (216 complete days of actigraphy data) | Mean activity count per minute in agitated vs non-agitated patients: 34.4 ± 22.5 vs 9.6 ± 12.2 (p<0.001); median Pearson correlation between daily RASS and activity counts = 0.43 |
| 38 | George BP | 2020 | Retrospective cohort study | Mechanically ventilated patients transported by air ambulance | Richmond Agitation-Sedation Scale (RASS) for sedation depth assessment | 327 patients | Adjusted relative risk (aRR) of hospital LOS for moderate sedation: 1.59 (95%CI 1.40–1.81); deep sedation aRR: 1.24 (95%CI 1.10–1.40); benzodiazepine associated with 2.9-day increase in hospital LOS (95%CI 0.7–5.1) |
| 39 | Lachs J | 2026 | Cross-sectional protocol analysis study | Publicly available statewide EMS protocols for agitated older adult patients (≥65 years) in the United States | Standardized protocol review tool with 26 evaluation elements | 34 state protocols + National EMS Model Guidelines | 97% of protocols included agitation management guidance; only 33% addressed older adults; 27% included sedative dose reduction for older adults; 82% included midazolam as a standing order |
| 40 | Olives TD | 2016 | Retrospective cohort study | Profoundly agitated adult patients treated with prehospital ketamine and transported to a Level 1 trauma center | Prehospital ketamine sedation protocol with EMS medical record review | 135 patients | Overall intubation rate = 63% (85/135); adjusted OR for intubation in males: 1.91 (95%CI 1.14–3.21); adjusted OR for overnight arrival: 2.57 (95%CI 1.05–6.27); no association between ketamine dose and intubation rate (p=0.68) |
| 41 | Jahromi MMH | 2026 | Randomized controlled trial (temporal cluster randomization) | Hemodynamically stable adult patients requiring ED-to-ICU transfer | Structured planned transfer protocol with continuous physiological monitoring, 4Score, Nonverbal Pain Scale (NVPS), RASS | 112 patients (56 in intervention group, 56 in control group) | Mean RASS post-transfer: -2.01 ± 1.61 (intervention) vs -2.64 ± 1.63 (control), p=0.04; transfer time: 9.4±2.0 min vs 16.0±4.0 min, p<0.01; no significant differences in physiological parameters, consciousness level or pain (all p>0.05) |

**Reference**

4.	Weeden M, Desai N, Sriram S, Swami Palaniswami M, Wang B, Talbot L, et al. A pilot study of high frequency accelerometry-based sedation and agitation monitoring in critically ill patients. Critical care and resuscitation : journal of the Australasian Academy of Critical Care Medicine. 2020;22(3):245-52. DOI: 10.1016/S1441-2772（23）00392-7

5.	Harsha MS, Bhatia PK, Sharma A, Sethi P. Comparison of Quantium Consciousness Index and Richmond Agitation Sedation Scale in Mechanically Ventilated Critically Ill Patients: An Observational Study. Indian J Crit Care Med. 2022;26(4):491-5. DOI: 10.5005/jp-journals-10071-24183

8.	Almeida TM, Azevedo LC, Nosé PM, Freitas FG, Machado FR. Risk factors for agitation in critically ill patients. Revista Brasileira de terapia intensiva. 2016;28(4):413-9. DOI: 10.5935/0103-507x.20160074

9.	Asmare TB, Diress GM, Gobezie NZ, Abuhay AG, Afework WA, Gedefaw GD, et al. Prevalence of Agitation and Its Associated Factors in Adult Intensive Care Unit: A Systematic Review and Meta-Analysis. Nurs Crit Care. 2026;31(1):e70296. DOI: 10.1111/nicc.70296

10.	Leong AY, Edginton S, Lee LA, Jaworska N, Burry L, Fiest KM, et al. Prevalence and incidence of ICU delirium and pain: a systematic review and meta-analysis. Intensive Care Med. 2025;51(11):2093-103. DOI: 10.1007/s00134-025-08167-7

11.	Seeger S, Dalyanoglu I, Nienhaus J, Yilmaz E, Castano LJV, Markser AM, et al. Delirium in critically ill COVID-19 patients: incidence, predictors, and outcomes in invasive mechanical ventilation with versus without ECMO support. J Cardiothorac Surg. 2026;21(1)DOI: 10.1186/s13019-026-04057-1

12.	Arroyo-Novoa CM, Figueroa-Ramos MI, Balas M, Rodríguez P, Puntillo KA. Opioid and Benzodiazepine Withdrawal Syndromes in Trauma ICU Patients: A Prospective Exploratory Study. Crit Care Explor. 2020;2(4):e0089. DOI: 10.1097/cce.0000000000000089

13.	Torres-Contreras CC, Páez-Esteban AN, Hinestrosa-Díaz Del Castillo A, Rincón-Romero MK, Amaris-Vega A, Martínez-Patiño JP. Factors associated with delirium in critical patients in a health institution in Bucaramanga, Colombia. Enfermeria intensiva. 2019;30(1):13-20. DOI: 10.1016/j.enfi.2018.03.002

14.	Kerber K, Zangmeister J, McNett M. Relationship Between Delirium and Ventilatory Outcomes in the Medical Intensive Care Unit. Critical care nurse. 2020;40(2):24-31. DOI: 10.4037/ccn2020697

15.	Bardwell J, Brimmer S, Davis W. Implementing the ABCDE Bundle, Critical-Care Pain Observation Tool, and Richmond Agitation-Sedation Scale to Reduce Ventilation Time. AACN advanced critical care. 2020;31(1):16-21. DOI: 10.4037/aacnacc2020451

16.	Qiu J, Meng Y, Yang Z, Ren R, Chen J, Huang H, et al. Associations of Intensive Care Unit Acquired Weakness and Postoperative Delirium in Surgical Intensive Care Unit: A Prospective Observation Study. Nurs Crit Care. 2025;30(3):e70061. DOI: 10.1111/nicc.70061

17.	Williams Roberson S, Azeez NA, Fulton JN, Zhang KC, Lee AXT, Ye F, et al. Quantitative EEG signatures of delirium and coma in mechanically ventilated ICU patients. Clinical neurophysiology : official journal of the International Federation of Clinical Neurophysiology. 2023;146:40-8. DOI: 10.1016/j.clinph.2022.11.012

18.	Moraes FDS, Marengo LL, Moura MDG, Bergamaschi CC, de Sá Del Fiol F, Lopes LC, et al. ABCDE and ABCDEF care bundles: A systematic review of the implementation process in intensive care units. Medicine. 2022;101(25):e29499. DOI: 10.1097/md.0000000000029499

19.	Kotfis K, Zegan-Barańska M, Żukowski M, Kusza K, Kaczmarczyk M, Ely EW. Multicenter assessment of sedation and delirium practices in the intensive care units in Poland - is this common practice in Eastern Europe? BMC Anesthesiol. 2017;17(1):120. DOI: 10.1186/s12871-017-0415-2

20.	Ho MH, Chang HR, Liu MF, Chen KH, Shen Hsiao ST, Traynor V. Recognizing Intensive Care Unit Delirium: Are Critical Care Nurses Ready? The journal of nursing research : JNR. 2022;30(3):e214. DOI: 10.1097/jnr.0000000000000487

21.	Nagaraj SB, Biswal S, Boyle EJ, Zhou DW, McClain LM, Bajwa EK, et al. Patient-Specific Classification of ICU Sedation Levels From Heart Rate Variability. Crit Care Med. 2017;45(7):e683-e90. DOI: 10.1097/ccm.0000000000002364

22.	Chanques G, Tarri T, Ride A, Prades A, De Jong A, Carr J, et al. Analgesia nociception index for the assessment of pain in critically ill patients: a diagnostic accuracy study. British journal of anaesthesia. 2017;119(4):812-20. DOI: 10.1093/bja/aex210

23.	Deutsch CK, Patnaik PP, Greco FA. Is There a Characteristic Autonomic Response During Outbursts of Combative Behavior in Dementia Patients? Journal of Alzheimer's disease reports. 2021;5(1):389-94. DOI: 10.3233/adr-210007

24.	Yu X, Hoog Antink C, Leonhardt S, Bollheimer LC, Laurentius T. Non-Contact Measurement of Heart Rate Variability in Frail Geriatric Patients: Response to Early Geriatric Rehabilitation and Comparison with Healthy Old Community-Dwelling Individuals - A Pilot Study. Gerontology. 2022;68(6):707-19. DOI: 10.1159/000518628

25.	Castro VM, Sacks CA, Perlis RH, McCoy TH. Development and External Validation of a Delirium Prediction Model for Hospitalized Patients With Coronavirus Disease 2019. J Acad Consult Liaison Psychiatry. 2021;62(3):298-308. DOI: 10.1016/j.jaclp.2020.12.005

26.	Davoudi A, Malhotra KR, Shickel B, Siegel S, Williams S, Ruppert M, et al. Intelligent ICU for Autonomous Patient Monitoring Using Pervasive Sensing and Deep Learning. Scientific reports. 2019;9(1):8020. DOI: 10.1038/s41598-019-44004-w

27.	Ramazi R, Bowen MEL, Flynn AJ, Beheshti R. Developing Acute Event Risk Profiles for Older Adults with Dementia in Long-Term Care Using Motor Behavior Clusters Derived from Deep Learning. Journal of the American Medical Directors Association. 2022;23(12):1977-83.e1. DOI: 10.1016/j.jamda.2022.04.009

28.	Enshaeifar S, Zoha A, Markides A, Skillman S, Acton ST, Elsaleh T, et al. Health management and pattern analysis of daily living activities of people with dementia using in-home sensors and machine learning techniques. PLoS One. 2018;13(5):e0195605. DOI: 10.1371/journal.pone.0195605

29.	Alam R, Bankole A, Anderson M, Lach J. Multiple-Instance Learning for Sparse Behavior Modeling from Wearables: Toward Dementia-Related Agitation Prediction. Annu Int Conf IEEE Eng Med Biol Soc. 2019;2019:1330-3. DOI: 10.1109/embc.2019.8856502

30.	Corradi JP, Thompson S, Mather JF, Waszynski CM, Dicks RS. Prediction of Incident Delirium Using a Random Forest classifier. Journal of medical systems. 2018;42(12):261. DOI: 10.1007/s10916-018-1109-0

31.	Sun H, Kimchi E, Akeju O, Nagaraj SB, McClain LM, Zhou DW, et al. Automated tracking of level of consciousness and delirium in critical illness using deep learning. NPJ digital medicine. 2019;2:89. DOI: 10.1038/s41746-019-0167-0

32.	Chua SJ, Wrigley S, Hair C, Sahathevan R. Prediction of delirium using data mining: A systematic review. J Clin Neurosci. 2021;91:288-98. DOI: 10.1016/j.jocn.2021.07.029

33.	Meerts S, D'Haese J, Vanhonacker D, Cools W, Ghijselings I. Comparison of NeuroSENSE ® and SedLine ® for measuring depth of sedation in the intensive care unit: an observational study: The PSIWAV study. Eur J Anaesthesiol Intensive Care. 2026;5(1):e0095. DOI: 10.1097/ea9.0000000000000095

34.	Dai PY, Wu YC, Sheu RK, Wu CL, Liu SF, Lin PY, et al. An automated ICU agitation monitoring system for video streaming using deep learning classification. BMC Med Inform Decis Mak. 2024;24(1):77. DOI: 10.1186/s12911-024-02479-2

35.	Mistraletti G, Umbrello M, Anania S, Andrighi E, A DIC, Martinetti F, et al. Neurological assessment with validated tools in general ICU: multicenter, randomized, before and after, pragmatic study to evaluate the effectiveness of an e-learning platform for continuous medical education. Minerva anestesiologica. 2017;83(2):145-54. DOI: 10.23736/s0375-9393.16.11103-4

36.	Walsh TS, Kydonaki K, Lee RJ, Everingham K, Antonelli J, Harkness RT, et al. Development of Process Control Methodology for Tracking the Quality and Safety of Pain, Agitation, and Sedation Management in Critical Care Units. Crit Care Med. 2016;44(3):564-74. DOI: 10.1097/ccm.0000000000001463

37.	Saavedra-Mitjans M, Van der Maren S, Gosselin N, Duclos C, Frenette AJ, Arbour C, et al. Use of actigraphy for monitoring agitation and rest-activity cycles in patients with acute traumatic brain injury in the ICU. Brain Inj. 2024;38(9):692-8. DOI: 10.1080/02699052.2024.2341323

38.	George BP, Vakkalanka JP, Harland KK, Faine B, Rewitzer S, Zepeski A, et al. Sedation Depth is Associated with Increased Hospital Length of Stay in Mechanically Ventilated Air Medical Transport Patients: A Cohort Study. Prehospital emergency care. 2020;24(6):783-92. DOI: 10.1080/10903127.2019.1705948

39.	Lachs J, Barghout R, Haussner W, Hancock D, Benton E, Dorsett M, et al. Exploring Current Emergency Medical Services Approaches to Manage Agitated Older Adult Patients: An Analysis of Statewide Protocols. J Am Geriatr Soc. 2026. DOI: 10.1111/jgs.70466

40.	Olives TD, Nystrom PC, Cole JB, Dodd KW, Ho JD. Intubation of Profoundly Agitated Patients Treated with Prehospital Ketamine. Prehosp Disaster Med. 2016;31(6):593-602. DOI: 10.1017/s1049023x16000819

41.	Jahromi MMH, Ravari A, Mirzaei T, Kamiab Z, Paghaleh ZR. Effect of planned in-hospital transfer on physiological indicators, level of consciousness, pain, and restlessness in Iran. Acute Crit Care. 2026. DOI: 10.4266/acc.004175
